# Correction: Venkatesan et al. A Perspective on Newly Emerging Proteolysis-Targeting Strategies in Antimicrobial Drug Discovery. *Antibiotics* 2022, *11*, 1717

**DOI:** 10.3390/antibiotics14121237

**Published:** 2025-12-08

**Authors:** Janarthanan Venkatesan, Dhanashree Murugan, Loganathan Rangasamy

**Affiliations:** 1 Drug Discovery Unit (DDU), Centre for Biomaterials, Cellular, and Molecular Theranostics (CBCMT), Vellore Institute of Technology (VIT), Vellore 632014, Tamil Nadu, India; janarthanan.v2022@vitstudent.ac.in (J.V.); dhanashree.murugan2019@vitstudent.ac.in (D.M.); 2 School of Advanced Sciences (SAS), Vellore Institute of Technology (VIT), Vellore 632014, Tamil Nadu, India; 3 School of Biosciences and Technology (SBST), Vellore Institute of Technology (VIT), Vellore 632014, Tamil Nadu, India

## Error in Figure/Table

In the original publication, there was a mistake in Tables 1 and 4 and Figure 7 as published. The errors in Tables 1 and 4 and Figure 7 were elaborated as follows: The corrected Tables 1 and 4 and Figure 7 appear below. Protac MyFit, mentioned in Table 1, is not a PROTAC-based therapy, but a proprioceptive-tactile stimulating vest. This information was added mistakenly to the table.

We kindly request you to remove the last row, S. No.14, in Table 1, and also the reference number 45.

1. Upon verifying the entire arrangements of tables, references, and the compound numbers, we found that the,

Table 4, S. No.2., the reference number, [62] (Morreale, F.E.; Kleine, S.; Leodolter, J.; Ovchinnikov, S.; Kley, J.; Kurzbauer, R.; Hoi, D.M.; Meinhart, A.; Hartl, M.; Haselbach, D.; et al. BacPROTACs Mediate Targeted Protein Degradation in Bacteria. *Cell* **2022**, *185*, 2338–2353.e18.) to be cited instead of the reference [64].

Table 4, S. No.2., Research outcome column, the compound was misnumbered, kindly change the text to, “JQ1(S) BacPROTAC, compound 50 showed the maximum degradation efficiency”.

2. In the ChemDraw image (Figure 7), the bonds are distorted and not visible; we kindly request that the original image be replaced with the one in the attached file.

The corrected figure has been attached.

**Figure 7:**
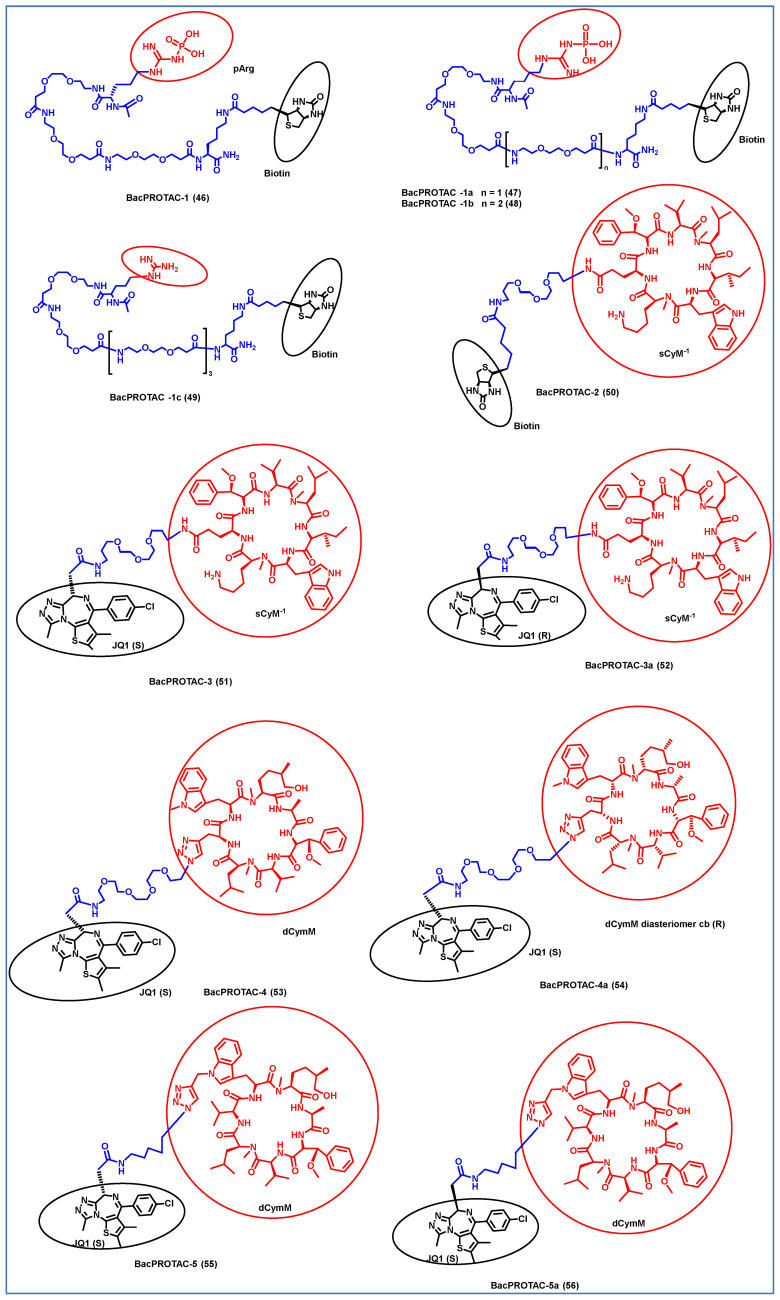
Structures of the PROTAC molecules are used for the degradation of proteins in prokaryotic cells using a bacterial proteolysis system. The red circle indicates the POI ligand. The Blue wavy line indicates the linker, and the black circle indicates the E3 ligand moiety.

## Missing Citation

In the original publication, reference 69 cited was not linked to the original reference. The citation, 69 in Patent Analysis and Future Perspectives, is to be updated.

Upon checking the references, we found that reference 69 is not cited in the particular patent, so we kindly request that the citation be changed to the reference given below. Kindly change the reference citation (69) to [69]. The available reference in the manuscript did not refer to the patent.

[69] Zhou, H.; Wu, S.; Xu, Z. A kind of Oseltamivir PROTAC Compound and its Preparation Method and Application in Anti-Influenza Virus Drug. CN112592331B, 22 October 2021. Available online: https://patents.google.com/patent/CN112592331B/en (accessed on 17 November 2022).

With this correction, the order of some references has been adjusted accordingly. The authors state that the scientific conclusions are unaffected. This correction was approved by the Academic Editor. The original publication has also been updated.
